# Does the angle of trocar insertion affect the fascial defect caused? A porcine model

**DOI:** 10.1007/s10029-023-02952-3

**Published:** 2024-02-06

**Authors:** C. Paasch, J. Meyer, R. Hunger, N. Krollmann, S. Heisler, R. Mantke

**Affiliations:** 1 Department of General and Visceral Surgery, University Hospital Brandenburg an der Havel, Brandenburg Medical University, Clinic for General and Visceral Surgery, Hochstraße 29, 14770 Brandenburg, Germany; 2 Department of General and Visceral Surgery, Ameos Hospital Schönebeck, Schönebeck, Germany; 3 Faculty of Health Sciences Brandenburg, Brandenburg Medical School Theodor Fontane, Brandenburg, Germany; 4 Faculty of Medicine, Brandenburg Medical School Theodor Fontane, Brandenburg, Germany

**Keywords:** Porcine model, Trocar site hernia, Bladed trocar, Bladeless trocar, Incisional hernia, Angle of trocar insertion, Conical trocar

## Abstract

**Introduction:**

With an incidence of 0–5.2%, trocar site hernias frequently occur following laparoscopy. It is unclear to what extent the angle of trocar insertion affects the size of the fascial defect caused. Hence, we performed a porcine model.

**Methods:**

In October 2022, a total of five female pigs were euthanized. In alternating order, three bladeless and two bladed conical 12-mm trocars were inserted at an angle of 45° on each side for 60 min twice each pig. For this purpose, an epoxy resin handmade cuboid with a central channel that runs at an angle of 45° was used. Subsequently, photo imaging and defect size measurement took place. The results were compared with those of our previously conducted and published porcine model, in which the trocars were inserted at an angle of 90°. Effects of trocar type (bladed vs. bladeless) and angle on defect size were analyzed using a mixed model regression analysis.

**Results:**

The bladeless trocars caused statistically significant smaller defects at the fascia than the bladed (23.4 (SD = 16.9) mm^2^ vs. 41.3 (SD = 14.8) mm^2^,* p* < 0.001). The bladeless VersaOne trocar caused the smallest defect of 16.0 (SD = 6.1) mm^2^. The bladed VersaOne trocar caused the largest defect of 47.7 (SD = 10.5) mm^2^. The defect size of the trocars used at a 45° angle averaged 30.5 (SD = 18.3) mm^2^. The defect size of trocars used at a 90° angle was significantly larger, averaging 58.3 (SD = 20.2) mm^2^ (*p* = 0.007).

**Conclusion:**

When conical 12-mm trocars are inserted at a 45° angle, especially bladeless ones, they appear to cause small fascial defects compared with insertion at a 90° angle. This might lead also to a lower rate of trocar hernias. Bladeless trocars might cause smaller fascial defects than bladed trocars.

**Supplementary Information:**

The online version contains supplementary material available at 10.1007/s10029-023-02952-3.

## Introduction

Trocar hernias frequently occur after laparoscopies. The inserted trocars lead to fascial defects and subsequently cause this postoperative late complication. The gynecologist Robert Fear from the United States first described these hernias in 1968 after performing a diagnostic laparoscopy [1].

A systematic review by Helgstrand et al. (2011) including 22 studies with 31,666 individuals revealed an incidence of 0–5.2% after laparoscopy with a substantially higher incidence when using trocars of > 10 mm compared with smaller trocars [2]. The majority occurred within 6 months after surgery.

Aside from the later appearance of these hernias and the problems they then cause (pain, nausea, and discomfort), fascial defects can also lead to early complications [2]. To that, Kwon et al. (2022) published a case report of a 25-year-old woman. She had a small bowel loop protruding through a 12-mm trocar defect in the right lower quadrant 48 h after the laparoscopic removal of the right tuba [3].

The size, and shape of the trocars, as well as the angle of insertion, might have an impact on the rate of trocar hernias. It has been frequently discussed [2, 4]. But evidence on that topic is low. Hence, current guidelines do not state which trocar design or angle of insertion should be used to prevent trocar hernias [5].

In 2022, we published the findings of a porcine model (*n* = 10) comparing the fascial defect size of conical bladed and bladeless trocars [6]. The trocars were inserted at an angle of 90°. These bladed and bladeless 12-mm conical trocars did not differ in terms of caused fascial defect size. We postulated that the occurrence of a trocar site hernia might be largely independent of trocar type. After analyzing the data and based on our clinical experience, we elaborate on the hypothesis that a more acute angle of trocar insertion may result in smaller defects.

Therefore, we performed the present pig model using the same experimental setup as in 2022, except that the trocars were placed at a 45° angle.

## Methods

In October 2022, the porcine model at hand was conducted in Beichlingen (Association for the Promotion of Innovative Medicine, Altenbeichlinger Str. 157, 99625, Beichlingen, Germany). According to §7 of the German Animal Welfare Act, the killing of an animal is not considered an animal experiment if the killing is done exclusively to use the animal’s organs or tissues for scientific purposes [7]. Hence, permission for an animal experiment was not obtained. No ethical approval was needed.

**Fig. 1:**
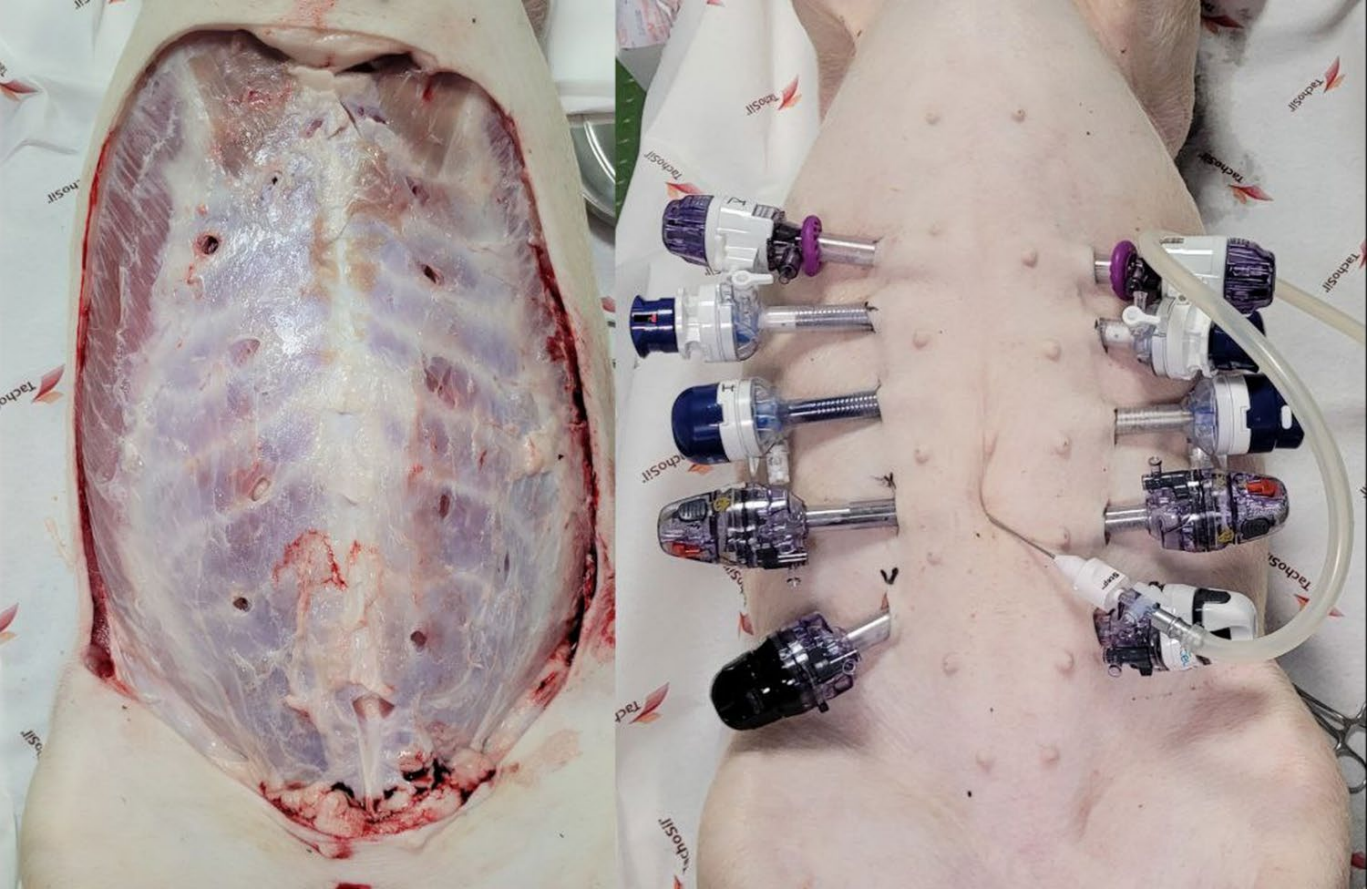
Different conical 12-mm trocar systems inserted at a 45° angle on each side 4 cm lateral of the mammary ridge

**Fig. 2:**
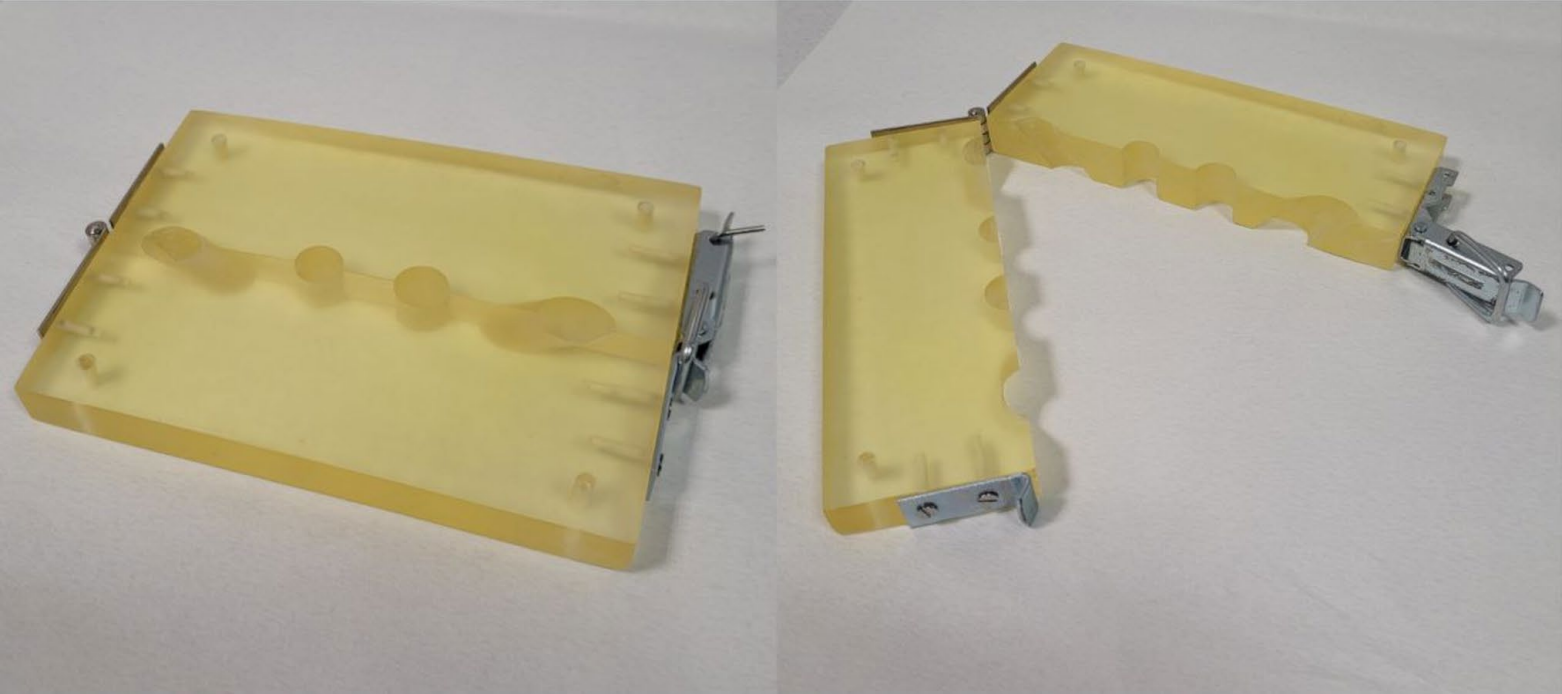
Handmade epoxy resin cuboid, 20 × 200 × 145 mm

A total of five female pigs (weight 32.6 kg (± 2.6); average age 90 ± 5 days) were euthanized with the short-acting barbiturate pentobarbital. With a Veress needle, a 12-mmHg pneumoperitoneum was conducted. Then in alternating order, five different conical 12-mm trocar systems were inserted at a 45° angle on each side 4 cm lateral of the mammary ridge (Fig. 1). Each trocar system was placed ten times (2/animal; Fig. S1). For this purpose, an epoxy resin cuboid was handmade (20 × 200 × 145 mm). This has a central channel that runs at an angle of 45° (Fig. 2). The insertion was performed with repetitive right quarter turning. A distance of 5 cm was maintained between two trocars. Their removal took place after 60 min. After trocar removal, the surrounding skin and fat tissue were removed with a scalpel. A ruler was then placed next to each fascial defect and photographs were taken. GSA Image Analyzer (v3.9.6, 2014) was used for defect measurement (mm^2^).

Two surgeons (experience > 10 years) inserted the trocars.

The following 12-mm bladed trocar systems were used: Bladed VersaOne (Covidien; B12STF conical tip) and ENDOPATH XCEL (Ethicon Endo-Surgery; D12LT conical tip).

The following 12-mm bladeless trocar systems were inserted: Bladeless VersaOne (Covidien; NONB12STF conical tip), ENDOPATH XCEL (Ethicon Endo-Surgery; B12LT conical tip), and Kii Fios First Entry (Applied Medical; CFF73 conical tip).

### Statistical analysis

Defect size was reported descriptively and stratified by trocar system using the mean (standard deviation), median (interquartile range, IQR), and range. As defect sizes were roughly normally distributed for each trocar system, we initially compared mean defect size between trocar systems using an ANOVA, ignoring the repeated measurement design. A significant main effect of trocar type on defect size was investigated further using pairwise Welch *t *tests with correction for multiple comparisons using the false discovery rate (FDR). For effect size, we computed omega squared (*ω*^2^) for the ANOVA main effect and Cohen’s d for pairwise comparisons.

To account for repeated measurements (10/animal), we fitted a linear mixed model using restricted maximum likelihood estimation (nloptwrap optimizer). The trocar system and the angle of insertion were entered as fixed and animal as random effects. In addition, an interaction between trocar system and angle was entered into the model. We hypothesized that there should be no significant main effect of trocar system. In a second model, angle and trocar type (bladed vs. bladeless) instead of the trocar system, as well as the interaction, were incorporated to model the defect size.

Statistical analysis was performed with R (R Software Foundation) and lme4 [8]. Model significance was examined by comparing the analytic model against the null model that only incorporates the intercept and the random effect of animal. Model performance was assessed using the marginal Nakagawa *R*^*2*^ and the residual standard deviation (sigma). As analysis of random effects is not of interest, the unadjusted intraclass correlation coefficient (ICC) was used to assess the importance of the grouping structure on the outcome, that is, how much the repeated measurement of the same animal impacted the defect size. Post hoc tests were performed using the emmeans-package, with Kenward-Roger approximation and an FDR adjustment [9]. Model comparisons were performed using likelihood ratio tests if models were nested, or Chi-square statistics if models with different fixed effects were compared.

A *p* value less than 0.05 was considered statistically significant. All tests were two-sided.

Due to the exploratory study design and lack of published studies on the effect of angle of insertion on defect size, a priori sample size calculation was not performed.

In the previously published pig model, all trocars were inserted at a 90° angle. Except for the angle, the previous animal experiment was set up the same way with the same number of animals (*n* = 10; average weight 37.85 kg; average age 90 ± 5 days). This data sheet was used to compare caused fascial defects after trocar insertion at 45° and 90° angles [6].

### Mixed model analysis for trocars inserted at angles of 45° and 90°

In the mixed linear model, not only the trocar system but also the angle and the interaction between angle and trocar type were investigated with regard to their effect on defect size. As per the previous model, the animal was considered as a random effect. With a comparably low unadjusted ICC (0.01), conditional *R*^2^ (0.46) and marginal *R*^2^ (0.45) are slightly higher. The *q* value column reports the adjusted *p *values (FDR).

## Results

The defect size of all trocars was on average 30.5 (18.3) mm^2^. Table 1 summarizes the defect sizes of every inserted trocar system. The bladeless VersaOne trocar caused the smallest defect of 16.0 (SD = 6.1) mm^2^. The bladed VersaOne trocar caused the largest defect of 47.7 (SD = 10.5) mm^2^. The results of the ANOVA revealed a significant main effect of trocar type on defect size (*F*(4, 45) = 5.854, *p* < 0.001, *ω*^2^ = 0.28). Three significant and substantial (large effect sizes) differences were observed, of which two are related to the smaller defect size of the bladeless VersaOne trocar. The defect size was smaller than for the bladed VersaOne trocar (adjusted *p* < 0.001, *d* = 3.69) and the bladed trocar ENDOPATH XCEL (adjusted *p* = 0.018, *d* = 1.55). The third significant difference was observed between the trocars Kii Fios First Entry bladeless and VersaOne bladed (adjusted *p* = 0.018, *d* = 1.48).

**Table 1:** Defect sizes of the inserted trocars

**Characteristic**	*n* = 50	**Endopath Xcel bladed** *n* = 10	**Endopath Xcel bladeless** *n* = 10	**Kii Fios First entry** *n* = 10	**VersaOne bladed** *n* = 10	**VersaOne bladless** *n* = 10
Defect size [mm^2^]						
Mean (SD)	30.5 (18.3)	34.9 (16.3)	29.3 (20.3)	24.8 (19.2)	47.7 (10.5)	16.0 (6.1)
Median (IQR)	24.8 (15.2–42.1)	32.2 (24.8–41.0)	25.2 (17.5–37.7)	18.1 (13.3–25.3)	44.3 (40.6–52.3)	15.9 (13.4–20.4)
Range	1.0, 67.2	14.4, 62.7	1.0, 64.8	7.6, 59.4	34.4, 67.2	6.6, 24.3

*IQR* interquartile range, *SD* standard deviation

**Fig. 3:**
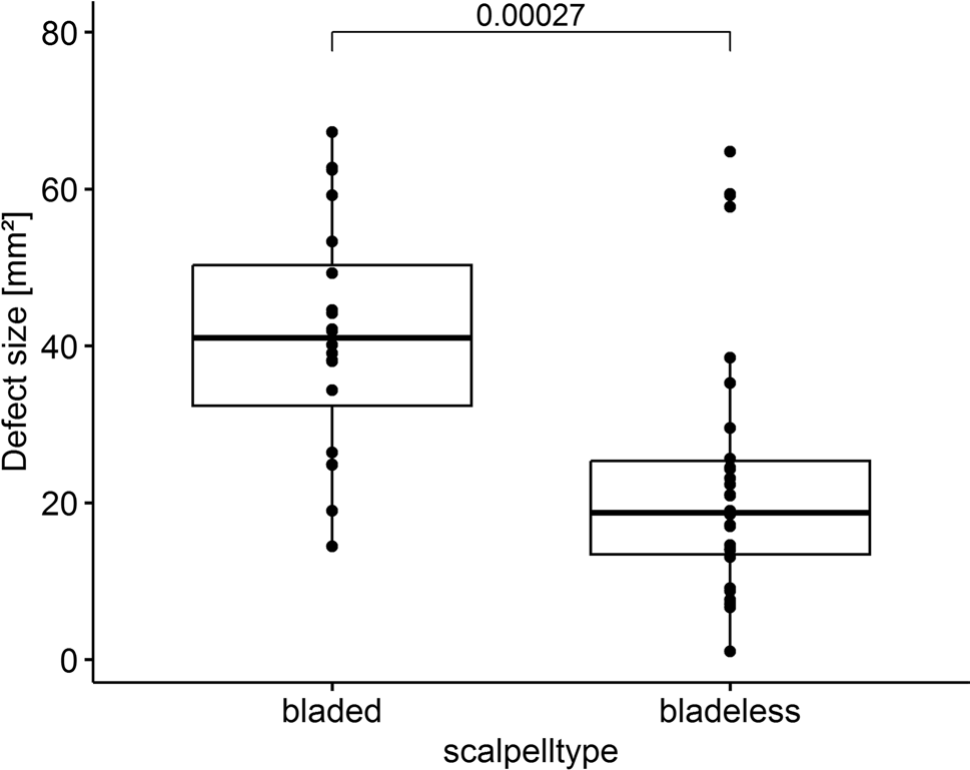
Comparison of defect sizes caused by all bladed and bladeless 12-mm trocar systems inserted at a 45° angle

The averaged defect size was 23.4 (16.9) mm^2^ when bladeless trocars were used. The trocars with blades resulted in a defect size of 41.3 (14.8) mm^2^. The bladeless trocars caused smaller defects on the fascia with statistical significance (*p* < 0.001; Figs. 3 and S2).

The five animals did not differ significantly with respect to defect size. The results are shown in Table S1.

### Descriptive comparison of trocars inserted at angles of 45° and 90°

The defect size of the trocars inserted at an angle of 45° was on average 30.5 (SD = 18.3) mm^2^. The defect size of the trocars placed at an angle of 90° was on average 58.3 (SD = 20.2) mm^2^.

In terms of the trocar system ENDOPATH XCEL bladed, the defect size averaged 34.9 (SD = 16.3) mm^2^ at a 45° angle and 58.1 (22.4) mm^2^ at a 90° angle. For the bladeless trocar ENDOPATH XCEL, the defect size averaged 29.3 (SD = 20.3) mm^2^ at a 45° angle and 55.0 (SD = 18.3) mm^2^ at a 90° angle. In terms of the bladeless Kii Fios First Entry trocar, the defect size averaged 24.8 (SD = 19.2) mm^2^ at a 45° angle and 69.3 (SD = 21.7) mm^2^ at a 90° angle. For the bladeless VersaOne trocar, the defect size averaged 16.0 (SD = 6.1) mm^2^ at a 45° angle and 54.1 (20.0) mm^2^ at a 90° angle. For the bladed VersaOne trocar, the defect size averaged 47.7 (SD = 10.5) mm^2^ at a 45° angle and 55.1 (SD = 18.4) mm^2^ at a 90° angle.

### Findings of the mixed model analysis for trocars inserted at angles of 45° and 90°

The analytical model performed significantly better than the null model (*χ*^2^ [df = 9] = 89.58, *p* < 0.001) and the model that incorporated the interaction term performed better than the model that was only based on the main effects (*χ*^2^ [df = 4] = 38.10, *p* < 0.001). The Nakagawa *R*^2^ marginal was 0.45 (95%-CI: 0.35, 0.57) and the residual standard deviation was 17.85 mm^2^. The effect of the repeated measurement on the same animal was practically zero (ICC unadjusted = 0.01), indicating that the animal did not affect the measured defect size. Accordingly, compared to the residual standard deviation, a low standard deviation of the random effect was estimated (SD = 2.48 mm^2^).

**Fig. 4:**
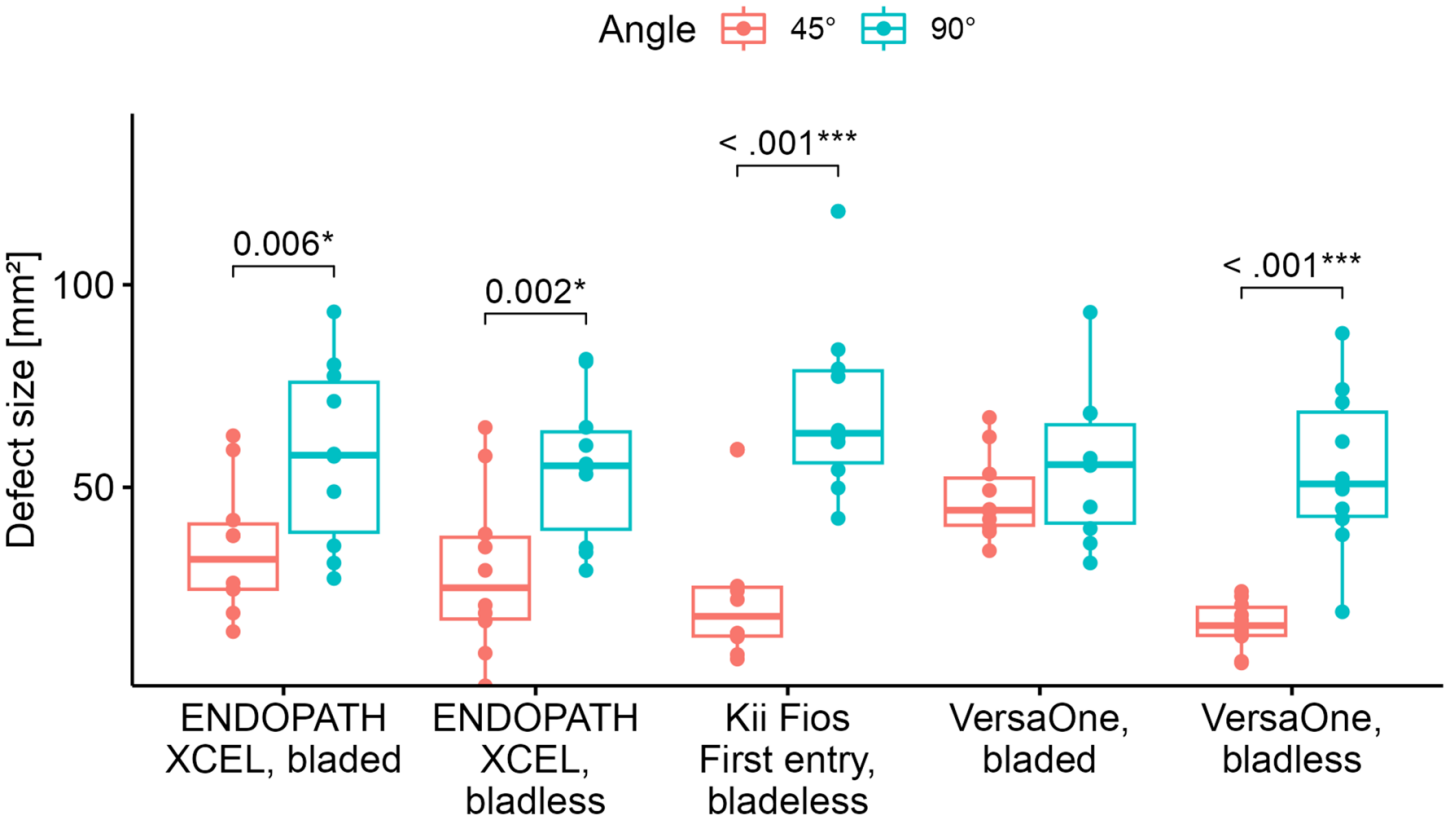
Comparison of defect sizes caused by trocar systems inserted at 45° and 90° angles

Results of the first model are summarized in Table 2. The intercept of 34.9 mm^2^ corresponds to a predicted defect size caused by a bladed ENDOPATH XCEL trocar inserted at an angle of 45°. The same trocar (bladed ENDOPATH XCEL) inserted at a 90° angle caused 23.2 mm^2^ larger defects, corresponding to a significant difference (adjusted *p* = 0.007). A significant effect was also observed for the trocar system (adjusted *p* = 0.005). The interaction between trocar system and angle was also significant (adjusted *p* = 0.012). The interaction terms can be interpreted as a trocar system-specific effect on defect size when the insertion angle is 90° instead of 45°, which is additional to the bladed ENDOPATH XCEL-specific difference of 23.2 mm^2^. So, for example, the bladeless VersaOne trocar is estimated to cause a 15.9 mm^2^ defect size (34.9–19.0 mm^2^), when inserted at an angle of 45°. However, when inserted at a 90° angle, the estimate is 54.0 mm^2^ (34.9–19.0 + 23.2 + 14.9 mm^2^). For results of post hoc tests, see Fig. 4.

**Table 2:** Mixed model analysis of trocars inserted at an angle of 45° and 90°

Characteristic	Beta	95% CI^a^	*q* value^b^
Intercept	34.9	23.5, 46.4	< 0.001
Type of trocar			0.005
Endopath Xcel bladed	–	–	
Endopath Xcel bladeless	– 5.7	– 21.6, 10.2	
Kii Fios First entry, bladeless	– 10.1	– 26.0, 5.8	
VersaOne bladed	12.7	– 3.2, 28.6	
VersaOne bladeless	– 19.0	– 34.9, – 3.1	
Angle			0.007
45°	–	–	
90°	23.2	7.0, 39.4	
Type of trocar* angle			0.012
Endopath Xcel bladeless * 90°	2.6	– 19.9, 25.0	
Kii Fios First entry * 90°	21.3	– 1.2, 43.7	
VersaOne bladed * 90°	– 15.8	– 38.3, 6.7	
VersaOne bladeless * 90°	14.9	– 7.6, 37.4	

Beta mm^2^

^a^CI = confidence Interval

^b^False discovery rate corrected *p* value for multiple testing

The results of the second mixed model that used the trocar-type factor (bladed vs. bladeless) instead of the trocar system factor (in combination with angle and an interaction between the two) are depicted in Table S2. The model resulted in reduced model performance (*χ*^2^ [df = 6] = 16.65, *p* = 0.01) compared to the first model. The model showed a significant main effect of angle (*P* < 0.001) and trocar type (*P* = 0.04) on defect size. The interaction term between trocar type and angle also gained significance (*P* = 0.036). Predicted defect sizes of the model are smallest for the 45°/bladeless combination (25.8 mm^2^), followed by the 45°/bladed combination (32.0 mm^2^). Predicted defect sizes for the angle of 90° are generally higher, independently whether bladed (57.6 mm^2^) or bladeless (72.3 mm^2^) trocar types were used.

## Discussion

The present porcine model aimed to provide further insight into the effects of the angle of trocar insertion on the size of the fascial defect. Research on that topic is mandatory to lower the rate of trocar hernias.

After mixed model analysis for trocars inserted at angles of 45° and 90°, we revealed that at an angle of 45°, the caused fascial defect was smaller with significance [6]. That seems to be surprising. Inserting a 12-mm trocar at an angle of 90° would dilate an area of at least 176.72 mm^2^ (semi-major axis: 7.5 mm). When the trocar is inserted at an angle of 45°, it has to dilate 249.92 mm^2^ of fascial tissue (Fig. S3). Somehow maybe the fascia ends come closer together after trocar removal when the trocar is inserted at an angle of 45°. But we do not have an explanation for that. On the other hand, it is comprehensible that trocar hernias occur less frequently when inserting them at an angle of 45° because of the backdrop phenomenon. The defect of the anterior rectus sheet is displaced relative to that of the posterior rectus sheet. But this backdrop phenomenon might be also a disadvantage. This will be the subject of further research. The group is currently preparing a test on human cadavers. The visualization of the fascia defect for suturing at the end of surgery might be more difficult. Laparoscopically assisted suturing could be a solution.

In addition, it has been recommended in the literature that defects of at least 10 mm should be sutured [2]. Thus, the majority of trocar hernias are likely to be due to forgotten or inadequate defect closure after trocar removal. The angle of insertion and the trocar shape may play a minor role in comparison.

Based on our findings, it seems that bladeless conical trocars inserted at an angle of 45° should be chosen for surgery. In contrast to our previous project, the bladeless trocars in this animal model caused smaller fascial defects than bladed trocars (bladed trocars: 41.3 (14.8) mm^2^; bladeless trocars: 23.4 mm^2^ (16.9; *p* = 0.00027). This difference in the effect of trocar type in relation to insertion angle was represented by the significant interaction term in the second mixed model. These results are consistent with the guideline recommendation that radially expanding blunt-tip trocars should be used in the repair of incisional hernias [10]. Bhoyrul et al. (1996) obtained similar results when they performed an animal model. Conventional bladed trocars and radially expanding bladeless trocars were used in laparoscopic cholecystectomies in 12 vivid pigs. The bladeless trocars also caused, with significance, a smaller defect size compared to the bladed trocars (approximately 50% narrower) [11]. Comparable results were also published in 2006 by Shafer et al. after they performed an animal experiment with eight vivid pigs. Trocars with blades caused larger defects than trocars without blades [12]. Contradictive results were published by Moreno et al. (2019) when operating on 11 vivid pigs [13]. But no information on the angle of insertion was stated in the three mentioned publications. Due to the different measurements, trocar shapes, and insertion techniques, these porcine models are only comparable to a limited extent. In addition, we operated on dead animals and not on live ones as in the three aforementioned publications. Unfortunately, it must be stated that in an unknown number of insertions in this porcine model (approximately twice), the blade was not removed with the provided button. However, this could mean that these trocars with blades would function like trocars without ones. The average defect size when inserting a bladed trocar at an angle of 45° could be even larger.

The VersaOne trocar without a blade caused the smallest defect of 16.0 (6.1) mm^2^. In the previous study, the VersaOne trocar without a blade also caused the smallest defect when inserted at a 45° angle (54.1 (20) mm^2^). In summary, this trocar caused the smallest defect in both animal models. But these findings cannot be confirmed when reviewing the literature. The aforementioned animal models did not use VersaOne trocars [4, 11, 12]. It remains unclear. But aside from the question of which trocar is best for preventing fascial defects and organ injury, we do not encourage the use of non-reusable trocars. Reusable devices are already widely used in routine surgery. The health sector must participate in environmental protection to a greater extent. Furthermore, in the United States, the United Kingdom, and Canada, healthcare produces 9.7 million tons of carbon annually [14]. Operating rooms are typically the most resource-intensive area of a hospital, 3–6 times more energy intensive than the rest of the hospital, and a major generator of waste [15].

It is known that the insertion of a trocar can cause organ injury, pain, and bleeding. [4, 16, 17]. An angle of 45° could reduce the risk of these complications since the depth of penetration is reduced.

In this project, we further developed the design and learned from the previous porcine model. We removed the abdominalis muscle, a thin muscle above the rectus abdominis muscle. We also made an epoxy cuboid with a 45° channel to ensure that the angle was always the same.

One study limitation is the fact that the experiment was performed on animals and not on humans. However, the anatomy is comparable, as the abdominal wall of pigs also consists of the obliquus externus, internus, and transversus abdominis muscles. Confirmation of our hypothesis that the angle of insertion affects the rate of trocar herniation in humans would be feasible but difficult. A trial on human cadavers is desirable and planned for the future. With an incidence of 0–5.2% after laparoscopy, a large sample size would be required for a randomized clinical trial. One option would be to collect data on the angle of insertion in existing hernia registries. A weakness of the present porcine model is also the fact that trocar insertion was performed by two surgeons. Only conical trocars were used. The subsequent defect measurement with the GSA Image Analyzer was not done by the same analyst as the previous project. On the other hand, there is no greater dispersion of values compared to the previous project (Fig. 4).

## Conclusion

When 12-mm conical trocars are inserted at a 45° angle, especially those without a blade, they appear to cause smaller fascial defects compared with insertion at a 90° angle. This could lead to a lower rate of trocar herniation.

Bladeless trocars might cause smaller fascial defects than bladed trocars.

### Supplementary Information

Below is the link to the electronic supplementary material.

Supplementary file1 Defect sizes in each animal (DOCX 13 KB)

Supplementary file2 Mixed model analysis of defect size by angle of insertion and trocar type (DOCX 14 KB)

Supplementary file3 Sequence of inserting the trocar (PNG 79 KB)

Supplementary file4 Comparison of defect sizes caused by each bladed and bladeless 12 mm trocar systems inserted at a 45° angle (PNG 124 KB)

Supplementary file5 Expected dilated area of fascia when 12 mm trocar systems were placed at 45° and 90° angles (JPG 40 KB)

## Data Availability

The data are available on reasonable request.
