# Supplementary material for: Does the angle of trocar insertion affect the fascial defect caused? A porcine model
Source: Hernia. 2024 Feb 6;28(2):585–92. doi: 10.1007/s10029-023-02952-3 (PMC10997682; doi:10.1007/s10029-023-02952-3)
Supplement: Supplementary file 1 — Supplementary file1 Defect sizes in each animal (DOCX 13 KB) [file 10029_2023_2952_MOESM1_ESM.docx]

**Table S1** Defect sizes in each animal

| **Characteristic** | n = 50 | **Animal I**  n = 10 | **Animal II**  n = 10 | **Animal III**  n = 10 | **Animal IV**  n = 10 | **Animal V**  n = 10 |
| --- | --- | --- | --- | --- | --- | --- |
| Defect size [mm²] |  |  |  |  |  |  |
| Mean (SD) | 30.5 (18.3) | 29.3 (20.8) | 35.3 (20.2) | 21.6 (13.0) | 32.8 (22.0) | 33.7 (13.8) |
| Median (IQR) | 24.8 (15.2 - 42.1) | 21.0 (14.1 - 40.5) | 30.6 (19.4 - 53.3) | 21.7 (10.3 - 26.0) | 32.1 (18.0 - 50.5) | 34.8 (24.6 - 38.4) |
| Range | 1.0, 67.2 | 8.7, 64.8 | 13.1, 67.2 | 6.6, 44.5 | 1.0, 62.7 | 14.0, 59.2 |

IQR interquartile range; SD standard deviation
