# Supplementary material for: Does the angle of trocar insertion affect the fascial defect caused? A porcine model
Source: Hernia. 2024 Feb 6;28(2):585–92. doi: 10.1007/s10029-023-02952-3 (PMC10997682; doi:10.1007/s10029-023-02952-3)
Supplement: Supplementary file 2 — Supplementary file2 Mixed model analysis of defect size by angle of insertion and trocar type (DOCX 14 KB) [file 10029_2023_2952_MOESM2_ESM.docx]

**Table S2** Mixed model analysis of defect size by angle of insertion and trocar type

| **Characteristic** | **Beta** | **95% CI**^1^ | **q-value**^2^ |
| --- | --- | --- | --- |
| Intercept | 69.3 | 57.3, 81.3 | < 0.001 |
| Angle |  |  | < 0.001 |
| 90° | — | — |  |
| 45° | -44.5 | -61.4, -27.6 |  |
| Trocar type |  |  | 0.040 |
| bladeless | — | — |  |
| bladed | -13.7 | -27.0, -0.5 |  |
| Interaction  45° x bladed | 20.9 | 2.1, 39.6 | 0.036 |
| Beta mm^2^  ^1^CI = Confidence Interval | | | |
| ^2^False discovery rate corrected p-value for multiple testing | | | |
