# Supplementary figures and images for: Does the angle of trocar insertion affect the fascial defect caused? A porcine model
Source: Hernia. 2024 Feb 6;28(2):585–92. doi: 10.1007/s10029-023-02952-3 (PMC10997682; doi:10.1007/s10029-023-02952-3)

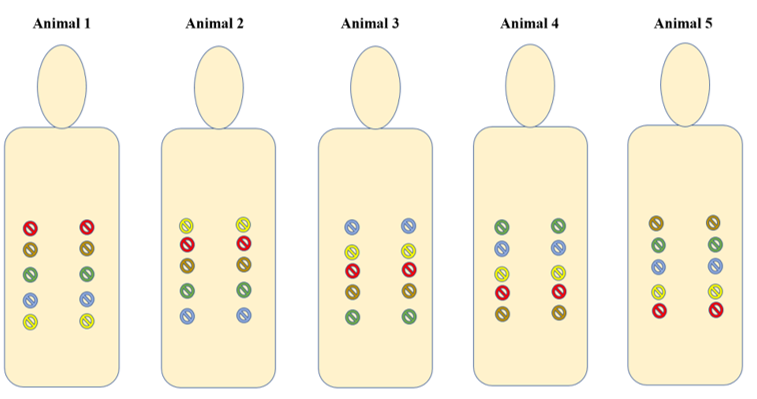

Supplement: Supplementary file 3 — Supplementary file3 Sequence of inserting the trocar (PNG 79 KB) [file 10029_2023_2952_MOESM3_ESM.png]

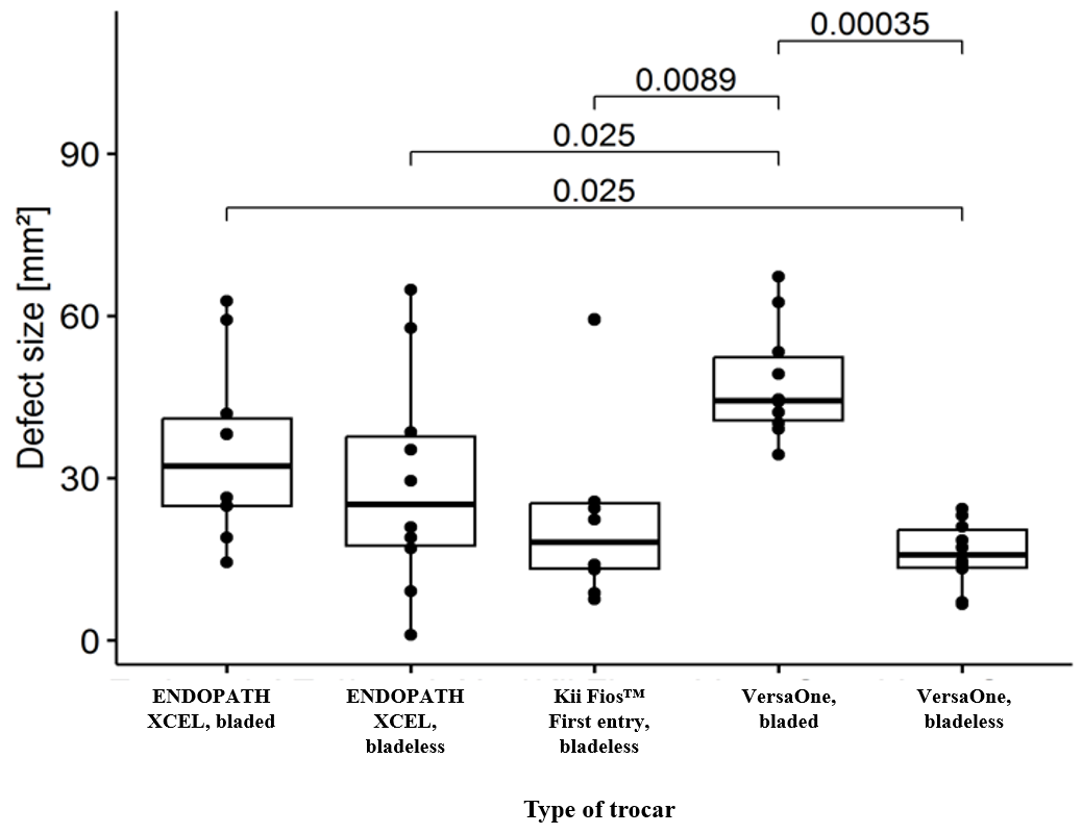

Supplement: Supplementary file 4 — Supplementary file4 Comparison of defect sizes caused by each bladed and bladeless 12 mm trocar systems inserted at a 45° angle (PNG 124 KB) [file 10029_2023_2952_MOESM4_ESM.png]

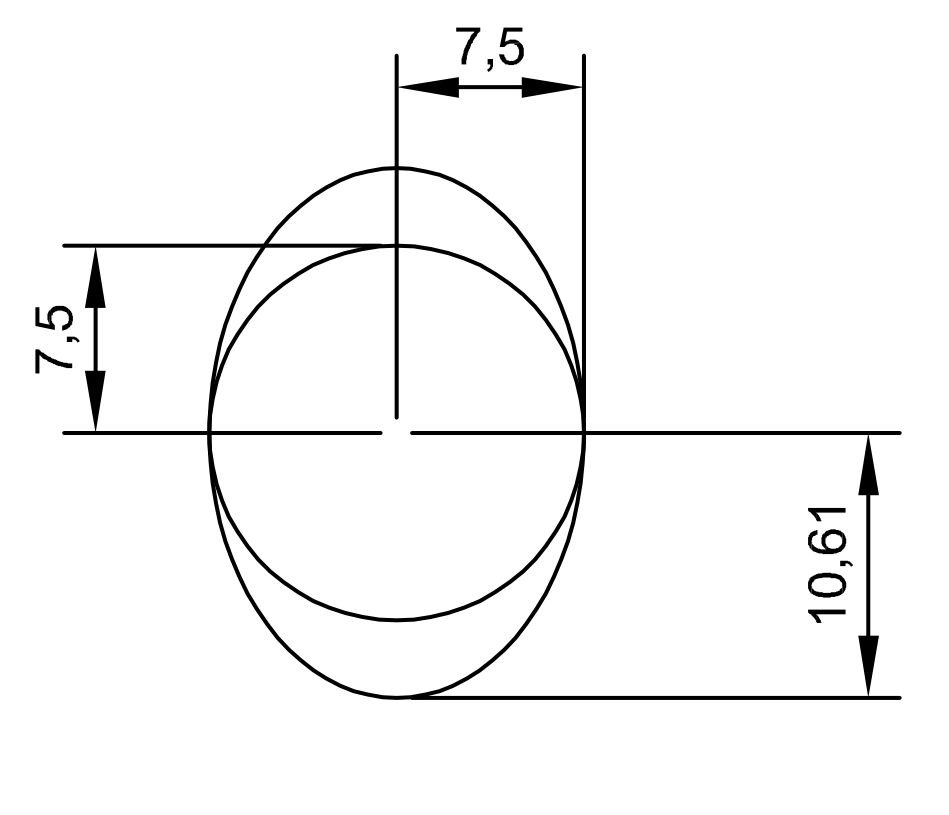

Supplement: Supplementary file 5 — Supplementary file5 Expected dilated area of fascia when 12 mm trocar systems were placed at 45° and 90° angles (JPG 40 KB) [file 10029_2023_2952_MOESM5_ESM.jpg]
